# Correction

**DOI:** 10.1111/cas.15030

**Published:** 2021-07-02

In an article^1^ titled “VPS33B interacts with NESG1 to suppress cell growth and cisplatin chemoresistance in ovarian cancer” by Yingxia Ning, Zhaoyang Zeng, Yuao Deng, Weifeng Feng, Lun Huang, Huiling Liu, Jiazhi Lin, Chen Zhang, Yue Fan, and Longyang Liu, the following errors were published:

- The first author’s affiliation was linked to the first and second affiliations in the published version, which is incorrect as she is only affiliated to ‘The First Affiliated Hospital of Guanzhou Medical University, Guanzhou, China’. The correct presentation is listed below.

Yingxia Ning^1^ | Zhaoyang Zeng^2^ | Yuao Deng^3^ | Weifeng Feng^4^ | Lun Huang^1^ | Huiling Liu^1^ | Jiazhi Lin^1^ | Chen Zhang^5^ | Yue Fan^6^ | Longyang Liu^7^

^1^Department of Gynaecology and Obstetrics, The First Affiliated Hospital of Guangzhou Medical University, Guangzhou, China

^2^Department of Gynecology and Obstetrics, The Integrated Hospital of Traditional Chinese Medicine, Southern Medical University, Guangzhou, China

^3^Department of Gynaecology and Obstetrics, Shenzhen People's Hospital, Shenzhen, China

^4^The First Affiliated Hospital of Jinan University, Guangzhou, China

^5^Department of Clinical Pharmacy, Guangzhou First People’s Hospital, Guangzhou, China

^6^Cancer Research Institute, Southern Medical University, Guangzhou, China

^7^Cancer Center, Integrated Hospital of Traditional Chinese Medicine, Southern Medical University, Guangzhou, China

- The authors would like to insert additional text in the Acknowledgments section and it should read:

**ACKNOWLEDGEMENTS**

We gratefully thank all of the authors for this study. This study was conducted both in “the First Affiliated Hospital of Guangzhou Medical University” and “Integrated hospital of Traditional Chinese Medicine, Southern Medical University”. Thanks for the help of Integrated Hospital of Traditional Chinese Medicine, Southern Medical University.

The authors apologize for the errors.
